# Activation of P2Y1R impedes intestinal mucosa repair during colitis

**DOI:** 10.7150/ijbs.82302

**Published:** 2023-08-21

**Authors:** Qiuchan Huan, Jiao Peng, Yaoyao Chang, Qiansheng Zhang, Tianhang Xing, Danling Jiang, Wenke Chen, Xiangchun Shen, Zhaoxiang Bian, Haitao Xiao

**Affiliations:** 1 School of Pharmaceutical Sciences, Health Science Center, Shenzhen University, Shenzhen 518060, China.; 2 Department of Pharmacy, Peking University Shenzhen Hospital, Shenzhen, China.; 3 The State Key Laboratory of Functions and Applications of Medicinal Plants and The High Efficacy Application of Natural Medicinal Resources Engineering Center of Guizhou Province, School of Pharmaceutical Sciences, Guizhou Medical University, Guizhou, China.; 4 Department of Urology, Minimally Invasive Surgery Center, The First Affiliated Hospital of Guangzhou Medical University, Guangzhou, China.; 5 Department of Gastroenterology, Peking University Shenzhen Hospital, Shenzhen, China.; 6 School of Chinese Medicine, Hong Kong Baptist University, Kowloon, Hong Kong.

**Keywords:** inflammatory bowel disease, intestinal mucosa repair, P2Y1R, AMPK, gut microbiota

## Abstract

Delayed intestinal mucosal healing is one of the pathogenic bases for the recurrence of inflammatory bowel disease (IBD), but how the IBD inflammatory environment impedes intestinal mucosa repair remains unclear. Adenosine diphosphate (ADP) is an endogenous ligand of P2Y1R that is highly produced at sites of inflammation. We herein identify a novel role of ADP to directly facilitate inflammation-induced epithelial permeability, delay wound healing, and disrupt tight junction integrity, and we found that P2Y1R, a receptor preferentially activated by ADP, was significantly upregulated in the colonic mucosa of ulcerative colitis (UC) patients and in colonic epithelial cells of colitis mice. Inhibition of P2Y1R significantly increased the epithelial permeability, decreased the wound healing capacity, and impaired the tight junction integrity in TNF-α-challenged Caco-2 cells. In parallel, the same effects in promoting intestinal mucosa repair were observed in DSS-induced colitis in P2Y1R^-/-^ mice. Mechanistic investigation revealed that P2Y1R inhibition facilitated epithelial AMP-activated protein kinase (AMPK) phosphorylation and gut microbiota homeostasis reconstruction. Taken together, these findings highlight that P2Y1R activation plays an important role in impeding intestinal mucosa repair during colitis, and that P2Y1R is an attractive target for the therapy of IBD.

## Introduction

Inflammatory bowel disease (IBD) is a chronic and recurrent colonic inflammatory disorder characterized by diarrhea, bleeding, and severe abdominal pain. Although this disease is highly prevalent in Western countries, recent studies have shown that it is rising rapidly in Asia, including China, with the growing popularity of the Western lifestyle 1, 2. Thus, IBD has emerged as a worldwide public health concern. Generally, this disease contains two main subtypes: ulcerative colitis (UC) and Crohn's disease (CD). To date, the pathogenesis of this disease remains largely unexplored, and it cannot be cured. Recently, numerous basic and clinical studies have suggested that mucosal healing is an ideal endpoint for IBD therapy because, while conventional drugs, such as aminosalicylates, corticosteroids, and immunosuppressants, are effective in reducing colonic inflammation and achieving a certain duration of remission, their effects in promoting intestinal mucosa healing are limited. The mucosa is left in an incomplete healing condition that facilitating the pathogenic factors invading again, and subsequent inducing the relapse of IBD 3-6. Therefore, it is important to explore the pathomechanisms involved in intestinal mucosal barrier dysfunction to discover new strategies for the treatment of IBD.

It is well known that during the process of colonic inflammation, adenosine triphospate (ATP) and adenosine diphosphate (ADP) are released and accumulated at sites of inflammation as damage-associated molecules to activate inflammatory events by binding to purinergic P2X and P2Y receptors 7, 8. A recent study determined ATP and ADP concentrations in colon tissues of dextran sulfate sodium (DSS)-induced colitis mice and found that ATP and ADP concentrations in inflamed colon tissues increased dramatically, with ADP being more than 66 times that of ATP 9. P2Y1R is a G protein-coupled receptor that mainly mediates the effect of ADP and is widely expressed in human and rodent immune cells, smooth muscle cells, enteric glial cells, and intestinal epithelial cells (IECs) 2, 7, 9-11. A series of previous studies have documented that P2Y1R plays a vital role in the pathogenesis of gastrointestinal disorders, such as IBD and irritable bowel syndrome (IBS). For instance, Hockley et al. reported that P2Y1R is a colonic nociceptor that could mediate the signal of visceral hypersensitivity and abdominal pain 12, and that inhibiting P2Y1R could reduce the visceral hypersensitivity of experimental IBS 13. Hwang et al. discovered that P2Y1R is one of the enteric motor regulators and that the loss of P2Y1R could slow colonic transit 14. Zhang et al. found that P2Y1R is an important molecule in the regulation of innate immunity, which can activate the NLRP3 inflammasome to aggravate experimental colitis 9. Our recent study found that P2Y1R is also an important molecule in the regulation of adaptive immunity, which can facilitate Th17 differentiation and promote colonic inflammation 2. However, the role of P2Y1R in intestinal barrier homeostasis remains unclear. In this study, we report that ADP, an endogenous ligand of P2Y1R, directly hinders the healing of colonic mucosal wounds and the recovery of epithelial barrier function during colitis. Crucially, we demonstrated that P2Y1R was upregulated in the colon epithelial tissues of UC patients and colonic epithelial cells of DSS-treated colitis mice, and the inhibition of P2Y1R promoted intestinal mucosa repair, which was associated with epithelial AMPK activation and gut microbiota homeostasis reconstruction.

## Materials and methods

### Human colon samples

Paraffin-embedded colon sections from active UC patients and healthy subjects were collected from the Department of Gastroenterology, Peking University Shenzhen Hospital (Shenzhen, China). Table 1 shows the corresponding clinical data for the selected healthy subjects and UC patients. The patients' colon tissue samples used in this study were approved by the institutional medical ethics committee (Ethics No. PN-202200128).

### Animals

P2Y1R knockout (P2Y1R^-/-^) mice were generated from MARC, Nanjing University (Model Animal Research Center, Nanjing University, China), and C57BL/6 wild-type (WT) mice were acquired from Beijing Vital River Laboratory Animal Technology Co., Ltd. (Beijing, China). All mice were raised in specific pathogen-free animal facilities with a light/dark cycle for 12 h. Six- to eight-week-old male offspring of C57BL/6 WT mice and P2Y1R^-/-^ mice were selected and used for the experiments. All animal operations were approved by the Animal Ethics Committee of Shenzhen University (Shenzhen, China).

### Colitis induction and evaluation

Colitis was developed by drinking water containing 2% DSS for 7 days, and disease activity index (DAI) scores were determined using a combination of body weight, stool consistency, and stool bleeding, as per our previous description 15.

### Isolation of primary murine IECs

Primary murine IECs were isolated as previously described 16. In brief, the mouse colons were opened longitudinally and rinsed with cold PBS to thoroughly flush away the contents. Subsequently, the samples of colon tissues were minced; resuspended in a buffer of Hanks balanced salt solution (HBSS) containing 5 mM EDTA, 2 mM DTT, and antibiotics; and gently rotated at 37°C for 30 min. Next, IECs were obtained by filtration through a 70 μm cell filter and centrifugation at 2,000 rpm for 15 min at 4°C.

### Cell culture and transfection

Caco-2 cells were obtained from the ATCC (Manassas, VA, USA) and cultured routinely, as in a previous study 17. To knock down AMP-activated protein kinase (AMPK) expression in Caco-2 cells, siRNA targeting AMPKα1 (The sequence: Sense 5′-CCA UGA AGA GGG CCA CAA UTT-3′, antisense: 5′-AUU GUG GCC CUC UUC AUG GTT-3′; GenePharma, Shanghai, China) was used. Caco-2 cells were transfected with siRNA using GP-Transfect-Mate in a serum-free opti-MEM medium. After transfection for 24 h, the cells were collected for analysis.

### Permeability assessment

The FITC-dextran (MW: 4kDa, Sigma, USA) was used to assess the permeability *in vivo* and* in vitro,* as previously reported, with minor modifications 18. For *in vitro* permeability assessment, Caco-2 cells (2 × 10^4^ cells/well) were seeded in the upper chamber of a 24-well Transwell chamber and cultured for 6-9 days to form a monolayer of cells with stable transmission. During this procedure, the medium was changed daily. Subsequently, the cell monolayers were treated with ADP for 2 h or MRS2179 for 24 h and then stimulated with TNF-α (100 ng/mL, Sangon Biotech, C600021-0010) for 24 h. After washing twice with PBS, the apical chamber was incubated with 1 mg/mL FITC-dextran for 2 h, and the basolateral chamber medium was collected for fluorescence analysis. For *in vivo* permeability assessment, mice with colitis were fasted for 12 h and given FITC-dextran by gavage at 0.6 mg/g body weight before the end of the experiment. After treatment with FITC-dextran 3 h, the mice were anesthetized, and their retroorbital blood was collected for fluorescence analysis.

### Histological examination

For colonic pathologic evaluation, 4% paraformaldehyde-fixed and paraffin-embedded sections of colon tissues were stained with hematoxylin and eosin (H&E), and then observed and scored in a blinded fashion as described previously 19. To count colonic goblet cells, paraffin-embedded sections of colon tissues were also subjected to alcian blue staining, and alcian blue-positive vacuoles in the whole section were calculated and compared among the groups.

### Cell immunofluorescence

Caco-2 cells were cultured in slides and grown to confluency, and the cell monolayers were treated with MRS2179 and TNF-α (100 ng/mL) for 24 h. Subsequently, the cells were fixed with 4% paraformaldehyde and permeabilized with 0.1% Triton X-100. After blocking with 5% BSA, the cells were incubated with primary antibodies overnight and Alexa Fluor 488-conjugated secondary antibody in the dark. After staining the nuclei with DAPI, immunolabeling was imaged using a confocal laser microscope.

### Tissue immunofluorescence

Deparaffinized colon sections were first subjected to repair the antigen with citrate buffer. After treatment with the endogenous peroxidase blocking solution (Dako Corp., Carpinteria, CA) and then 10% goat serum to reduce nonspecific staining, the sections were incubated with primary antibodies overnight and Alexa Fluor 594-conjugated secondary antibody in the dark. After staining the nuclei with DAPI, the images were obtained using a fluorescence microscope (Zeiss, Oberkochen, Germany).

### Wound healing assay

A wound healing assay was conducted on Caco-2 cells, as described previously 20. In brief, Caco-2 cells were seeded in 12-well plates and left to grow to confluency, and then the cell monolayers were scratched using a 250 μL pipette tip. After washing with PBS, the cell monolayers were treated with ADP for 2 h or MRS2179 in the presence of TNF-α (100 ng/mL) for 24 h, and cellular migration was observed and captured under a microscope.

### Real-time qPCR analysis

Total RNA was extracted from cells or colon tissues with TRIzol® (Thermo Fisher, USA), and then transcribed to cDNA using a reverse transcription kit (TaKaRa, Shiga, Japan). Quantitative real-time PCR (qPCR) was performed using SYBR Green Master Mix (Roche Diagnostics GmbH Mannheim, Germany) on an ABI 7500 Real-Time PCR System, and mRNA expression was normalized with GAPDH control. **Table 2** lists the primer sequences used to amplify the targeting genes.

### Western blot (WB) analysis

WB analysis was performed as described previously 15. In brief, after quantification, proteins extracted from cells or colon tissues were separated by SDS-PAGE gels, transferred to nitrocellulose membranes, and blocked with 5% BSA. After incubation with primary antibodies and corresponding HRP-conjugated secondary antibodies, the protein bands were visualized using an ECL substrate (BIO-RAD, 1705061, Hercules, CA, USA). The primary antibodies used for the WB are listed as follows: GAPDH (Cell Signaling Technology, 2118S), P2Y1R (Affinity, DF10258), p-AMPK (Bioworld Technology, BS5003), AMPK (Bioworld Technology, BS1009), ZO-1 (Abcam, ab96587), occludin (Abcam, ab222691), and claudin-2 (Cell Signaling Technology, 48120).

### 16S rRNA gene high throughput sequencing

Fresh fecal samples from four groups of mice (WT + H_2_O, P2Y1R^-/-^ + H_2_O, WT + DSS, and P2Y1R^-/-^ + DSS) were collected, and their total genomic DNA was extracted with HiPure Soil DNA kits according to the manufacturer's instructions. Diluted DNA was then used to amplify the 16S rRNA V3-V4 region with barcoded primers (341F: CCTACGGGNGGCWGCAG, 806R: GGACTACHVGGGTATCTAAT). Subsequently, the PCR products were sequenced and analyzed, as previously reported 21.

### Short-Chain Fatty Acids (SCFA) Analysis

The levels of SCFA (acetic acid, propionic acid, and butyric acid) were measured using a 3-nitrophenylhydrazine derivatization strategy. About 100 mg of feces were extracted with 20 × 70% methanol (m/v) and then processed with TissueLyzer after adding zinc beads. The fecal samples were then centrifuged at 14,000 rpm at 4°C for 15 min. About 200 μL of upper supernatant was transferred to new tubes for LC-MS analysis. An Agilent 1290 Infinity II UPLC system coupled with a triple quadrupole 6470 mass spectrometer was used for targeted metabolomic profiling. A Waters BEH C_18_ column (50 mm × 2.1 mm, 1.7 μm) with a precolumn was used. The MS data were collected and processed using Agilent software. The chromatographic condition was used as described previously by Dei Cas et al 22, a and mass spectrometry detection was performed as described in our previous study 23.

### Statistical analysis

Statistical analysis was conducted using GraphPad Prism 6.0 software (GraphPad Software Inc., San Diego, CA, USA), and the results were presented as mean ± SEM. The tests used include unpaired Student's t-test and one-way ANOVA with Dunnett's test, depending on the dataset. A p-value < .05 was considered statistically significant.

## Results

### Extracellular ADP disturbs intestinal mucosa repair during colitis

**Figure 1:**
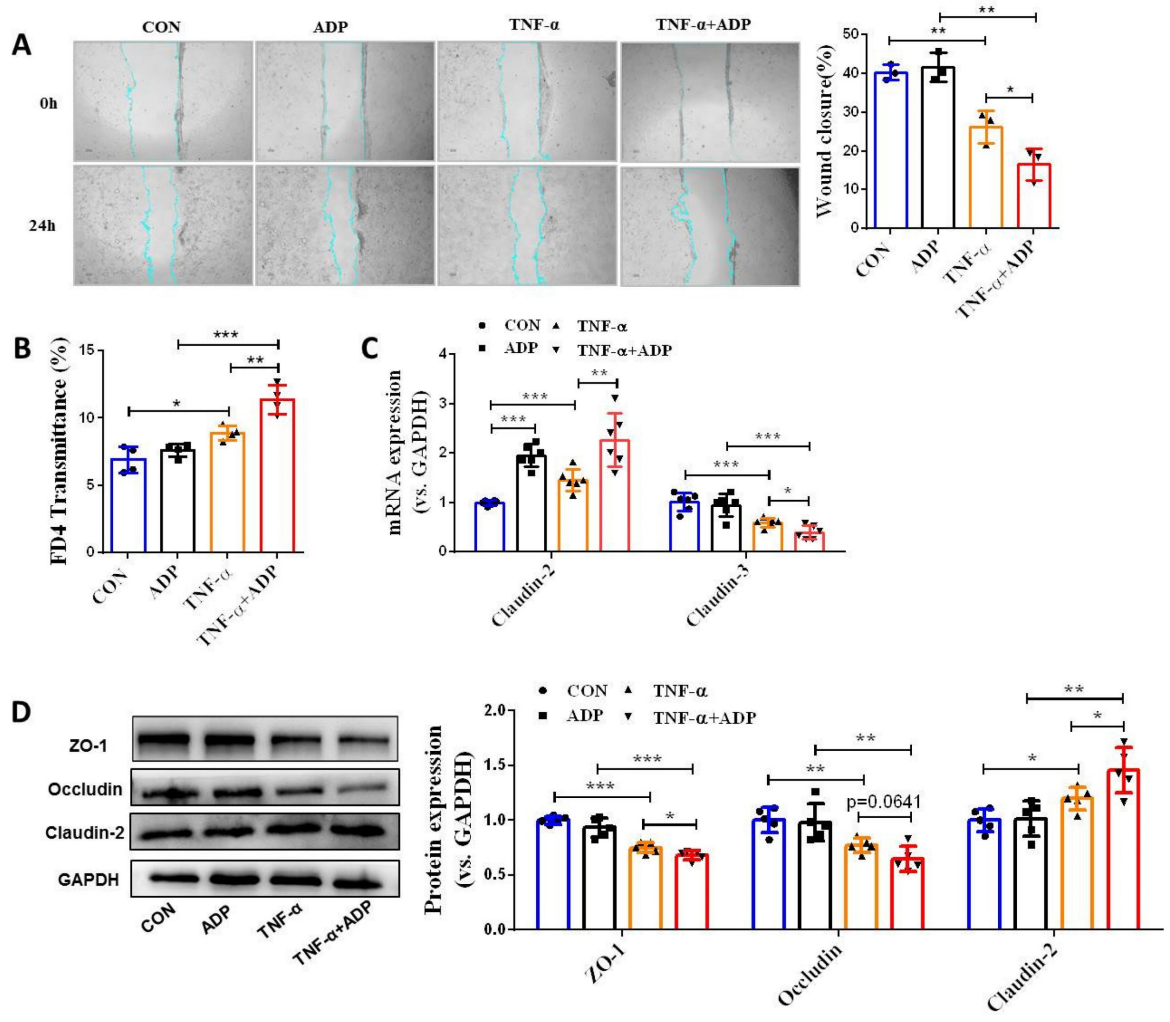
** Extracellular ADP disturbed intestinal mucosa repair under inflammatory conditions *in vitro*. (A)** Confluent Caco-2 monolayers were scratched and treated with ADP (1 mM) for 2 h and TNF-α (100 ng/mL) for 24 h. The wound closure was quantified and calculated. **(B)** Caco-2 monolayers with stable transmission were treated with ADP (1 mM) for 2 h and TNF-α (100 ng/mL) for 24 h, followed by incubation with 1 mg/mL FITC-dextran in the apical chamber for 2 h. Medium from the basal chamber was collected and analyzed for FITC-dextran transmission. **(C-D)** Caco-2 monolayers were treated with ADP (1 mM) for 2 h and TNF-α (100 ng/mL) for 24 h to detect the mRNA levels of claudin-2 and claudin-3 **(C)** and the protein levels of ZO-1, occludin, and claudin-2 **(D)**. Data represent the means ± SEM. *p < .05, **p < .01, ***p < .001.

**Figure 2:**
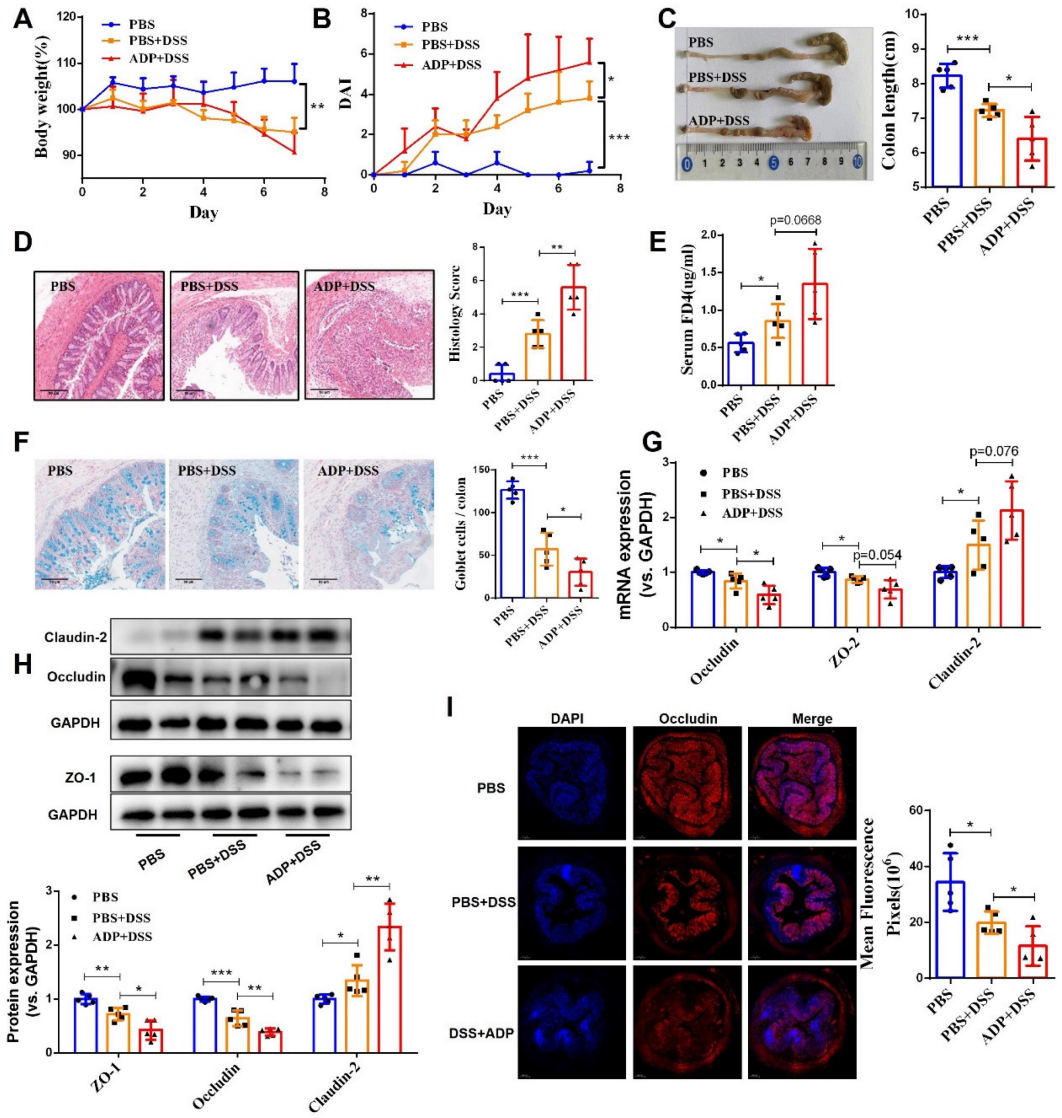
** Extracellular ADP aggravated intestinal epithelial barrier dysfunction in DSS-induced colitis mice.** Colitis was induced in WT C57BL/6J mice (n = 5) by daily administration of PBS or ADP (100 mg/kg) from days 1 to 7. At the end of the experiment, the mice were fasted for 12 h, and colonic permeability was measured by intragastric administration of FITC-dextran. (**A**) Body weight loss, and (**B**) DAI score. **(C)** Representative colon morphology and length. **(D)** Representative histological analysis images (× 100) and pathological score. **(F)** Representative images of alcian blue staining and goblet cell count. **(E)** Concentration of FITC-Dextran in mouse serum, and** (G)** mRNA expression of ZO-2, occludin, and claudin-2 in colon tissues.** (H)** Protein expression of ZO-1, occludin, and claudin-2 in colon tissues. **(I)** Immunofluorescent staining (× 200) of occludin (red) and DAPI (blue) for colon tissues, and quantitative analysis of fluorescence intensity. Data represent the means ± SEM. *p < .05, **p < .01, ***p < .001.

During colitis, both ATP and ADP dramatically increased at the site of inflammation, but the concentration of ADP was tens of times higher than that of ATP, since ATP can be rapidly hydrolyzed to produce ADP 9. Therefore, we first investigated the biological significance of ADP secretion on the intestinal mucosal barrier in the *in vitro* Caco-2 cell model under normal physiological and inflammatory conditions. As shown in **Figure 1**, ADP treatment had no significant effect on intestinal mucosal barrier function under normal physiological conditions, but only increased the mRNA level of claudin-2 **(Figure 1C)**. Unexpectedly, the increased cell monolayer permeability and decreased wound healing capacity in the TNF-α treated Caco-2 cell monolayer was significantly aggravated after ADP treatment** (Figure 1A** and** 1B)**. In addition, ADP treatment with significantly increasing claudin-2 expression and decreasing ZO-1, occludin and claudin-3 at gene and/or protein levels led to increased fragmentation monolayer tight junction integrity **(Figure 1C** and** 1D)**. Subsequently, we investigated the pathogenic effect of ADP on DSS-induced colonic inflammation in mice. Compared to the mice treated with DSS alone, DSS combined with ADP-treated mice exhibited more severe clinical manifestations in DAI scores (day 7), colon length shortening, and colon tissue damage **(Figure 2A-2D)**. Pathological examination also revealed that ADP plus DSS-treated mice had far fewer goblet cells **(Figure 2F)**. In addition, the *in vivo* permeability assessment showed that the concentration of FITC-Dextran in the serum of ADP plus DSS-treated mice was much higher than that of the mice treated with DSS alone **(Figure 2E)**. The results of the WB, RT-PCR, and immunofluorescence also showed that colon tissues from the mice treated with ADP plus DSS had higher levels of claudin-2 and lower levels of ZO-1, ZO-2, and occludin **(Figure 2G-2I)**. Collectively, these results demonstrate that ADP secretion during colitis directly hinders intestinal mucosa repair.

### P2Y1R expression is upregulated in patients with UC and IECs of DSS-induced colitis mice

**Figure 3:**
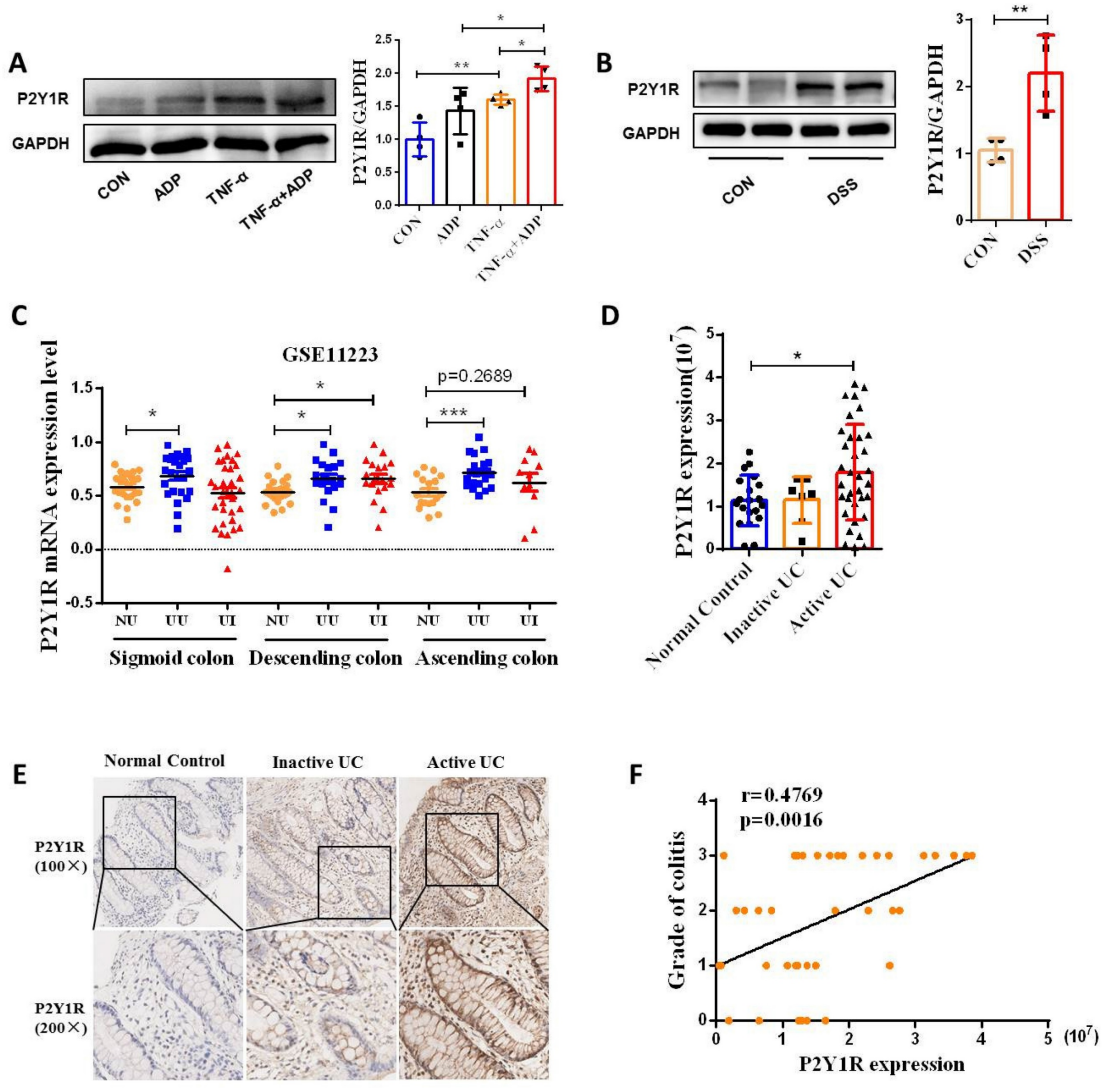
** P2Y1R expression was upregulated in the colonic epithelial cells of mice with DSS-induced colitis and in the colonic mucosa of UC patients. (A)** Caco-2 monolayers were treated with ADP (1 mM) for 2 h and TNF-α (100 ng/mL) for 24 h, and then P2Y1R levels were measured by WB. **(B)** WT C57BL/6J mice were treated with or without 2% DSS for 7 days. Primary colonic epithelial cells were isolated from the mice, and the level of P2Y1R was determined by WB. **(C)** P2Y1R mRNA levels in inflamed and noninflamed colonic mucosa tissues of UC patients were obtained from GSE11223. **(E)** Representative P2Y1R immunohistochemical staining (× 200) and **(D)** quantitative analysis of P2Y1R expression (20 cases of healthy subjects, seven cases of inactive UC patients, and 34 cases of active UC patients). **(F)** Spearman correlation between P2Y1R expression and endoscopic score of colitis (r = 0.4769, *p* =.0016). Data represent the means ± SEM. *p < .05, **p < .01, ***p < .001.

ADP, an endogenous ligand of P2Y1R, P2Y12R, and P2Y13R, preferentially activates P2Y1R in endothelial cells 24. Therefore, we detected the protein expression of P2Y1R in Caco-2 cell monolayers. As shown in** Figure 3A**, ADP treatment significantly increased the TNF-induced upregulation of P2Y1R expression. Next, we detected protein expression of P2Y1R in the IECs of colitis mice, and the same result was observed **(Figure 3B)**. To investigate whether the expression of P2Y12R is upregulated in the colon tissues of UC patients, we first used the microarray data GSE11223 downloaded from the Gene Expression Omnibus (GEO) database for analysis. Consistent with the results obtained from cell and animal experiments, the P2Y1R mRNA levels in inflammatory and non-inflammatory colonic mucosal tissues in patients with UC were much higher than in normal tissues of healthy controls, especially in colonic mucosal tissues from the descending colon of UC patients **(Figure 3C)**. Subsequently, we used the immunohistochemical (IHC) method to detect the expression of P2Y1R in colon tissue samples from UC patients. The results showed that P2Y1R was expressed in both the cytoplasm and nucleus of epithelial cells **(Figure 3E)**, and its expression was significantly increased during the disease's active phase **(Figure 3D)**. In addition, Spearman correlation analysis showed that P2Y1R expression in colon tissue was positively correlated with the severity of colitis **(Figure 3F)**. Overall, these data suggest that P2Y1R expression was significantly increased during colitis.

### P2Y1R inhibition promotes intestinal mucosa repair *in vitro* and *in vivo*

**Figure 4:**
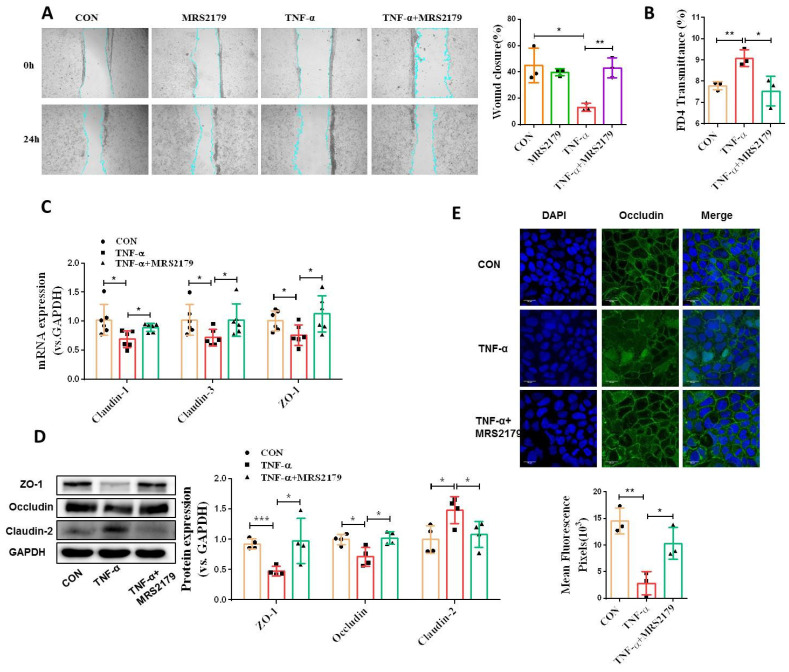
** P2Y1R inhibition promoted intestinal mucosa repair *in vitro*. (A)** Confluent Caco-2 monolayers were scratched and treated with MRS2179 (5 µM) and TNF-α (100 ng/mL) for 24 h. The wound closure was quantified and calculated. **(B)** Caco-2 monolayers with stable transmission were treated with MRS2179 (5 µM) and TNF-α (100 ng/mL) for 24 h, followed by incubation with 1 mg/mL FITC-dextran in the apical chamber for 2 h. Medium from the basal chamber was collected and analyzed for FITC-dextran transmission. **(C-E)** Caco-2 monolayers were treated with MRS2179 (5 µM) and TNF-α (100 ng/mL) for 24 h to detect the mRNA levels of claudin-1, claudin-3, and ZO-1 **(C)**, the protein levels of ZO-1, occludin, and claudin-2 **(D),** and the fluorescence intensity of occludin in immunofluorescent staining (× 200, occludin (green) and DAPI (blue)) **(E)**. Data represent the means ± SEM. *p < .05, **p < .01, ***p < .001.

**Figure 5:**
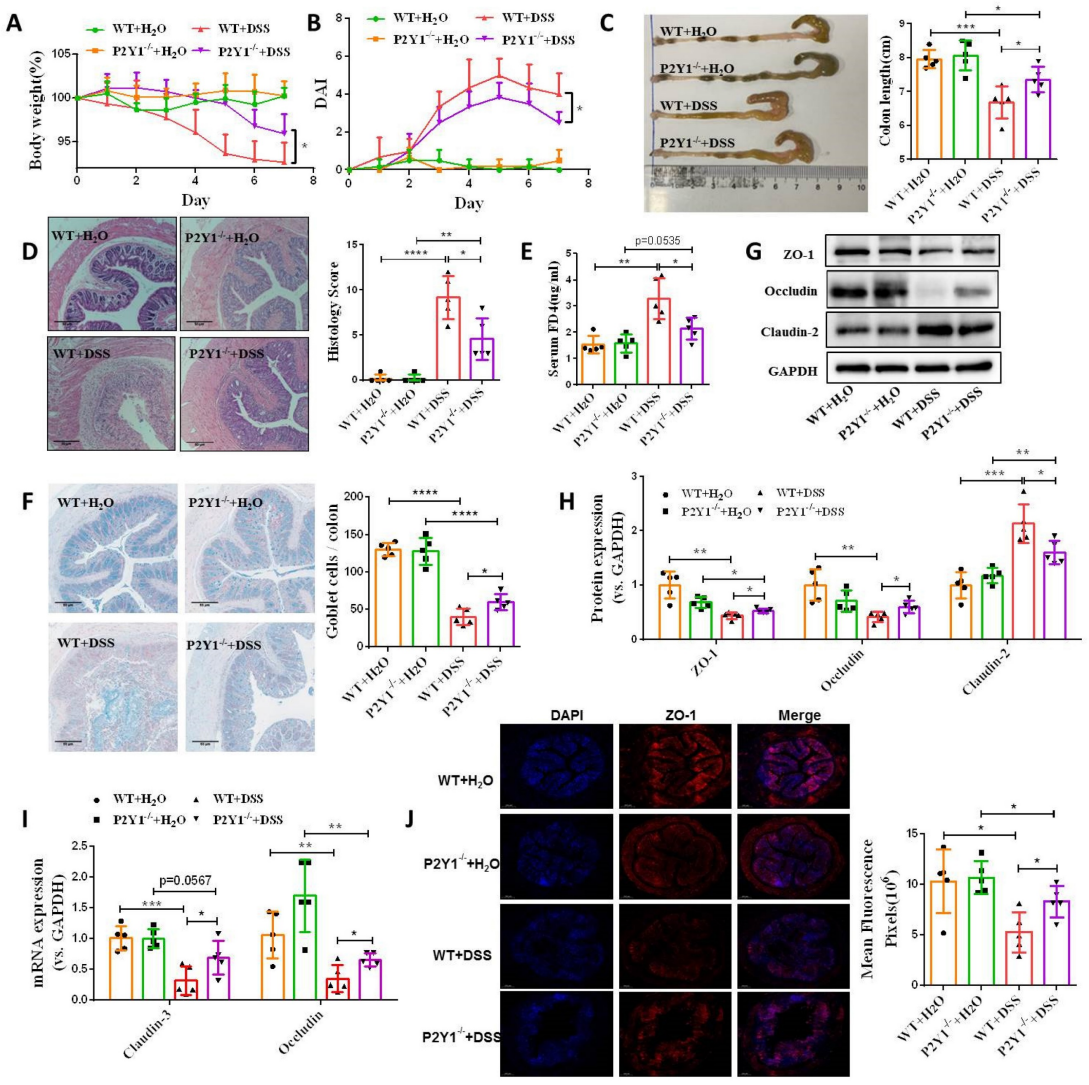
** P2Y1R inhibition promoted intestinal mucosa repair *in vivo*.** Colitis was induced in WT or P2Y1R^-/-^ C57BL/6J mice (n = 5) for 7 days. At the end of the experiment, the mice were fasted for 12 h and colonic permeability was measured by intragastric administration of FITC-dextran. (**A**) Body weight loss, and (**B**) DAI score. **(C)** Representative colon morphology and length. **(D)** Representative histological analysis images (× 100) and pathological score. **(E)** Concentration of FITC-Dextran in mouse serum. **(F)** Representative images of alcian blue staining and goblet cell count.** (G** and** H)** Protein levels of ZO-1, occludin, and claudin-2, and** (I)** mRNA levels of occludin and claudin-3 in colon tissues.** (J)** Immunofluorescent staining (× 200) of ZO-1 (red) and DAPI (blue) for colon tissues, and quantitative analysis of fluorescence intensity. Data represent the means ± SEM. *p < .05, **p < .01, ***p < .001.

To address the functional role of P2Y1R in intestinal mucosal repair during colitis, we next evaluated the effect of inhibiting P2Y1R with its specific antagonist, MRS2179, on TNF-α-treated Caco-2 cell monolayers. As shown in **Figure 4A** and** 4B**, TNF-α treatment significantly increased the permeability and decreased the wound healing capacity of cell monolayers, while these changes were greatly rescued after inhibiting P2Y1R. Moreover, inhibition of P2Y1R also significantly suppressed the decrease in TNF-α-induced tight junction proteins claudin-1, claudin-3, ZO-1, and occludin, and the increase in claudin-2 **(Figure 4C-4E)**. To further elucidate the role of P2Y1R inhibition in promoting intestinal mucosa repair *in vivo*, P2Y1R^-/-^ mice were used. Consistent with previous reports, P2Y1R^-^*^/^*^-^ mice were resistant to DSS challenge, showing less body weight loss (day 7), lower DAI score (day 7), longer colon length, and decreased pathological scores **(Figure 5A-5D)**. A permeability assay showed that the serum FITC-dextran concentration in P2Y1R^-/-^ colitis mice was significantly lower than that in WT colitis mice, suggesting that P2Y1R deficiency reduced intestinal permeability in colitis mice **(Figure 5E)**. In addition, alcian blue staining showed that the goblet cell number in P2Y1R^-/-^ colitis mice was significantly higher than that in the WT colitis mice **(Figure 5F)**. Consistent with the above results, tight junction integrity analysis also found that the levels of ZO-1, occludin, and claudin-3 were significantly upregulated, while the level of claudin-2 was significantly downregulated after P2Y1R deficiency **(Figure 5G-5J)**. Taken together, these results demonstrate that P2Y1R inhibition promotes intestinal mucosa repair.

### P2Y1R regulates epithelial AMPK activation *in vitro* and *in vivo*

**Figure 6:**
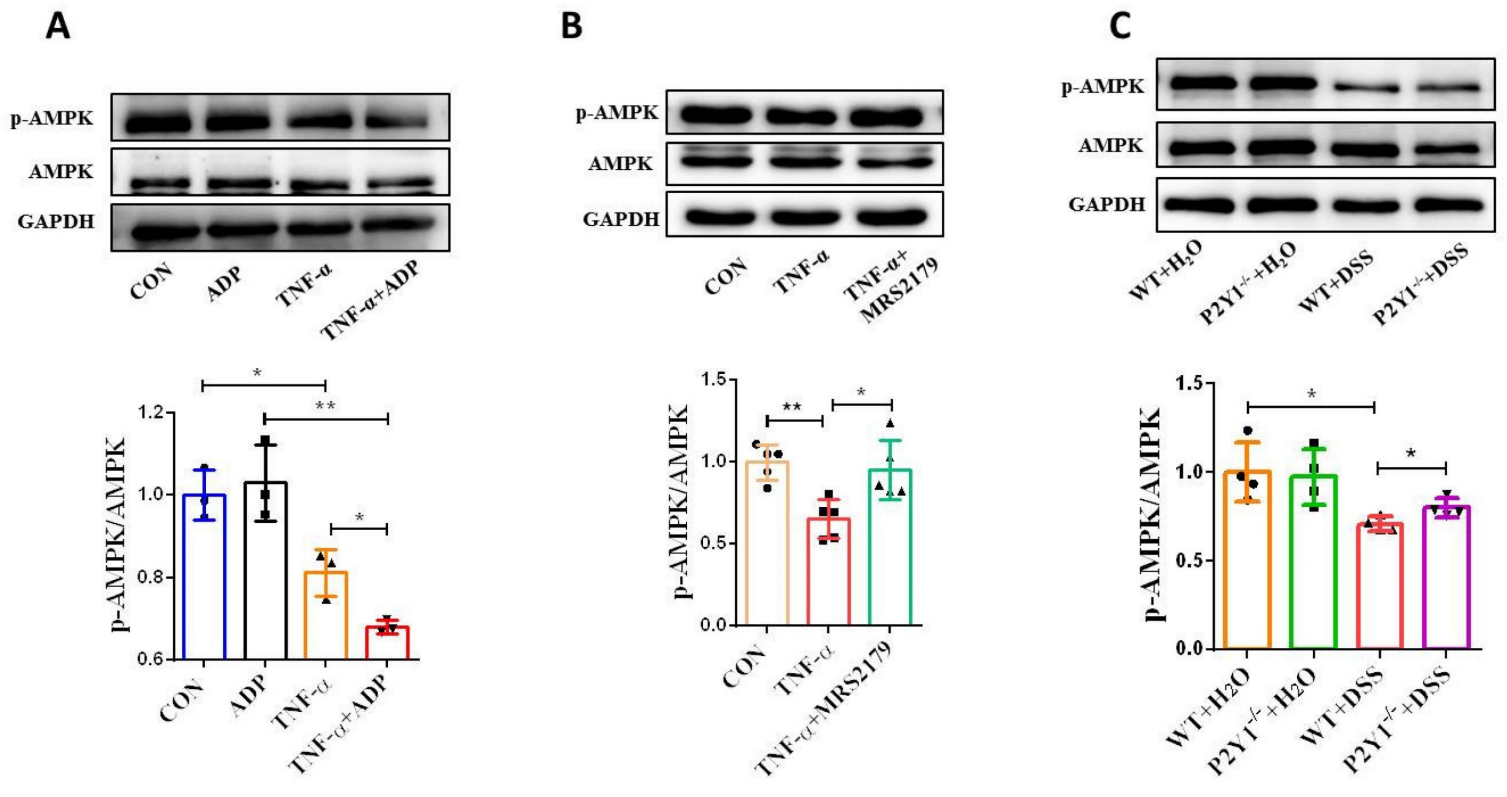
** P2Y1R regulated epithelial AMPK activation *in vitro* and *in vivo*.** Caco-2 monolayers were treated with ADP (1 mM) for 2 h or MRS2179 (5 µM) in the presence of TNF-α (100 ng/mL) for 24 h to detect p-AMPK levels by WB **(A** and** B)**. WT or P2Y1R^-/-^ C57BL/6J mice were treated with or without 2% DSS for 7 days, primary colonic epithelial cells were isolated, and then p-AMPK levels were determined by WB **(C)**. Data represent the means ± SEM. *p < .05, **p < .01.

AMPK, a crucial regulator of metabolic homeostasis, has recently been identified as a gatekeeper of the intestinal epithelial barrier, playing a key role in promoting epithelial differentiation and facilitating cell polarity establishment, stabilizing, and assembling tight junctions 25-28. We detected p-AMPK protein expression in TNF-α-treated Caco-2 monolayers and found significant inactivation. Notably, extracellular ADP treatment significantly promoted TNF-α-induced p-AMPK inactivation **(Figure 6A)**. However, treatment with the P2Y1R antagonist MRS2179 significantly inhibited TNF-α-induced p-AMPK inactivation **(Figure 6B)**. Subsequently, we examined p-AMPK expression in IECs isolated from P2Y1R^-/-^ and WT colitis mice. Consistent with the results of the *in vitro* experiments, the expression level of p-AMPK in WT colitis mice was lower than that in WT control mice, while the expression level of p-AMPK in P2Y1R^-/-^ colitis mice was higher than that in WT colitis mice **(Figure 6C)**. Collectively, these results suggest that P2Y1R regulates epithelial AMPK activation during colitis.

### Blocking AMPK activation impairs P2Y1R inhibition-mediated intestinal mucosa repair *in vitro* and *in vivo*

**Figure 7:**
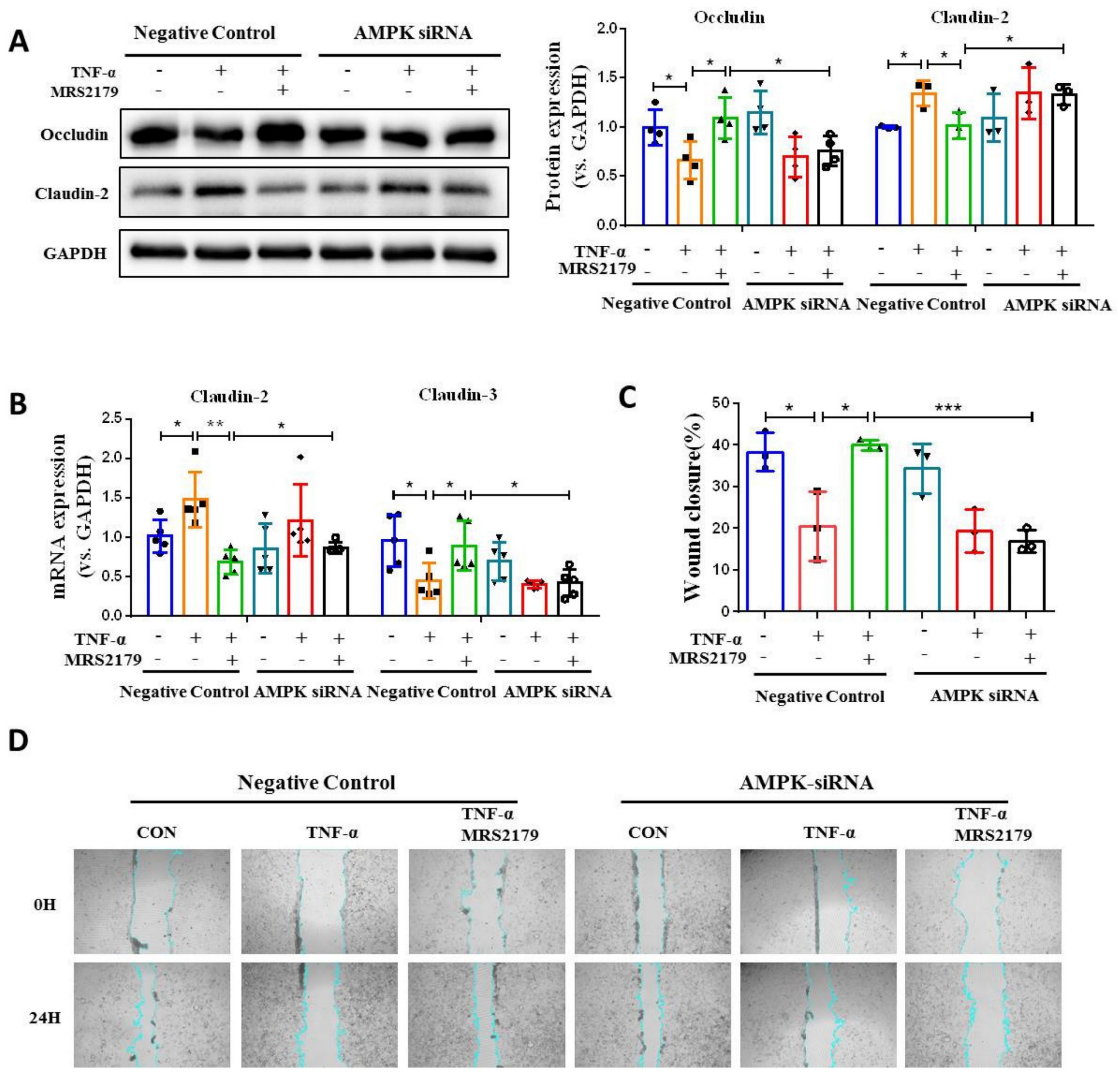
** Blockade of AMPK activation impaired intestinal mucosal repair mediated by P2Y1R inhibition *in vitro*.** (**A** and** B**) Transfected with AMPK-targeting siRNA Caco-2 cells or nontargeted siRNA Caco-2 cells were treated with MRS2179 (5 µM) and TNF-α (100 ng/mL) for 24 h to detect the protein levels of occludin and claudin-2 and the mRNA levels of claudin-2 and claudin-3. (**C** and** D**) Caco-2 cells were transfected with AMPK-targeted siRNA and nontargeted siRNA. After confluence, the monolayers were scratched and treated with MRS2179 (5 µM) and TNF-α (100 ng/mL) for 24 h. The wound closure was quantified and calculated. Data represent the means ± SEM. *p < .05, **p < .01, ***p < .001.

**Figure 8:**
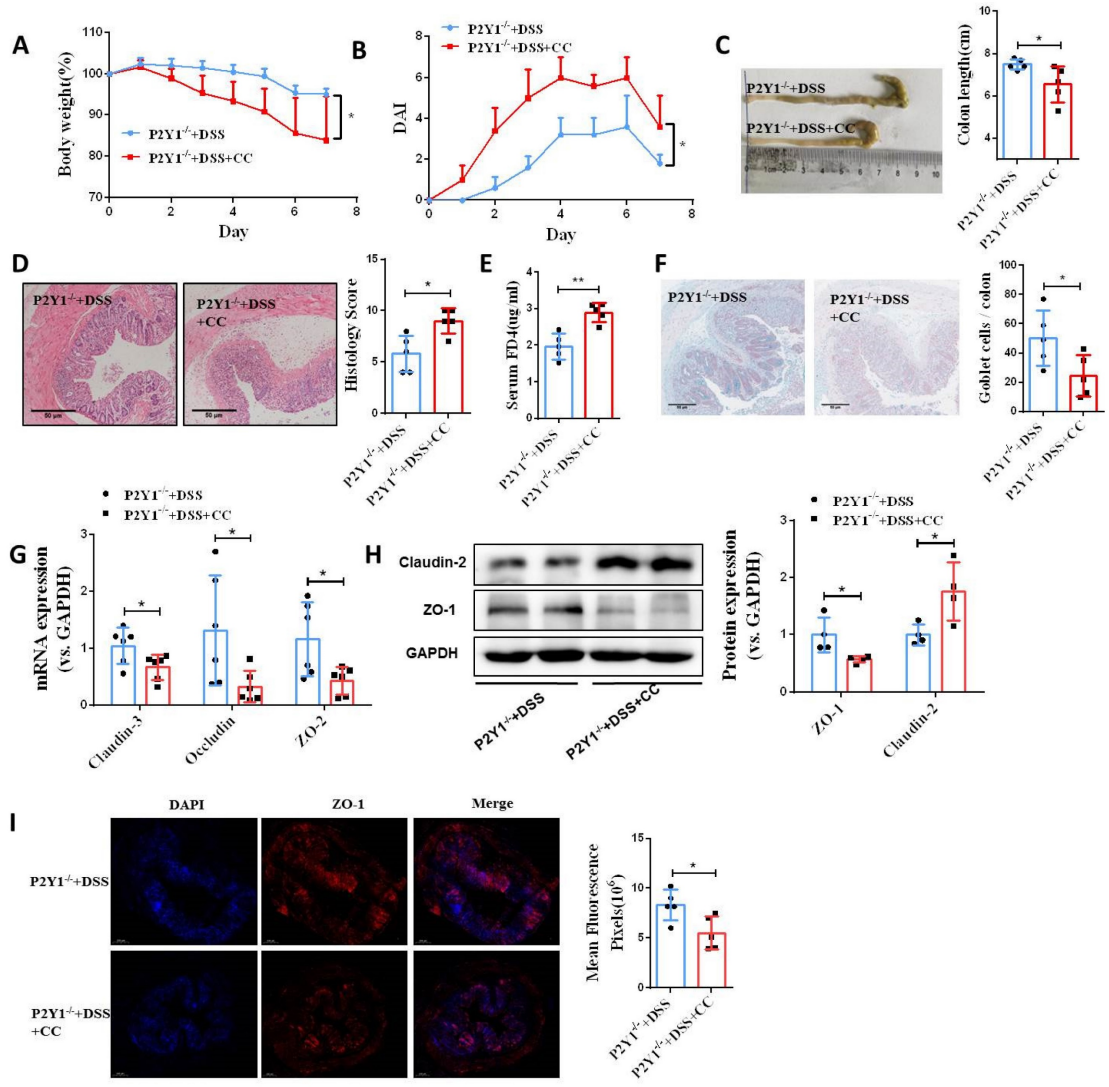
** Blockade of AMPK activation impaired intestinal mucosal repair mediated by P2Y1R inhibition *in vivo*.** Colitis was induced in P2Y1R^-/-^ C57BL/6J mice (n = 5) by daily administration of PBS or Compound C (CC, 10 mg/kg) from days 1 to 7. At the end of the experiment, mice were fasted for 12 h and colonic permeability was measured by intragastric administration of FITC-dextran. (**A**) Body weight loss and (**B**) DAI score. **(C)** Representative colon morphology and length. **(D)** Representative histological analysis images (× 100) and pathological score.** (E)** Concentration of FITC-Dextran in mouse serum. **(F)** Representative images of alcian blue staining and goblet cell count.** (G)** mRNA expression of ZO-2, occludin, and claudin-3 in colon tissues.** (H)** Protein expression of ZO-1 and claudin-2 in colon tissues. **(I)** Immunofluorescent staining (× 200) of ZO-1 (red) and DAPI (blue) for colon tissues, and quantitative analysis of fluorescence intensity. Data represent the means ± SEM. *p <.05, **p < .01.

Next, we investigated the role of AMPK activation in intestinal mucosa repair after P2Y1R inhibition. First, we used siRNA to silence AMPK signaling in Caco-2 cell monolayers treated with TNF-α and MRS2179. The results showed that the increase of tight junction proteins claudin-3 and occludin and the decrease of claudin-2 caused by P2Y1R inhibition were significantly reversed (**Figure 7A** and** 7B**). In addition, the promoting effect of P2Y1R inhibition on the wound healing of Caco-2 cell monolayers was significantly attenuated after AMPK blockade **(Figure 7C** and** 7D)**. We then blocked AMPK signaling in DSS-induced colitis in the mice using the AMPK antagonist Compound C (CC, 10 mg/Kg, i.p., Sigma-Aldrich) and found that Compound C greatly aggravated colitis in P2Y1R^-/-^ mice, as shown by increased weight loss (day 7), increased DAI score (day 7), a shortened colon, and colonic tissue damage **(Figure 8A-8D)**. In parallel, permeability assay and alcian blue staining demonstrated a significant increase in intestinal permeability and goblet cell loss in the colon after Compound C treatment **(Figure 8E** and** 8F)**. Consistent with these results, Compound C treatment significantly reduced the levels of tight junction proteins ZO-1, ZO-2, occludin, and claudin-3 and increased claudin-2 levels in colon tissues of P2Y1R^-/-^ colitis mice **(Figure 8G-8I).** Taken together, these results suggest that AMPK activation mediates the beneficial effects of P2Y1R inhibition in promoting intestinal mucosa repair.

### P2Y1R inhibition alters the composition of gut microbiota and the content of fecal SCFAs of DSS-induced colitis mice

**Figure 9:**
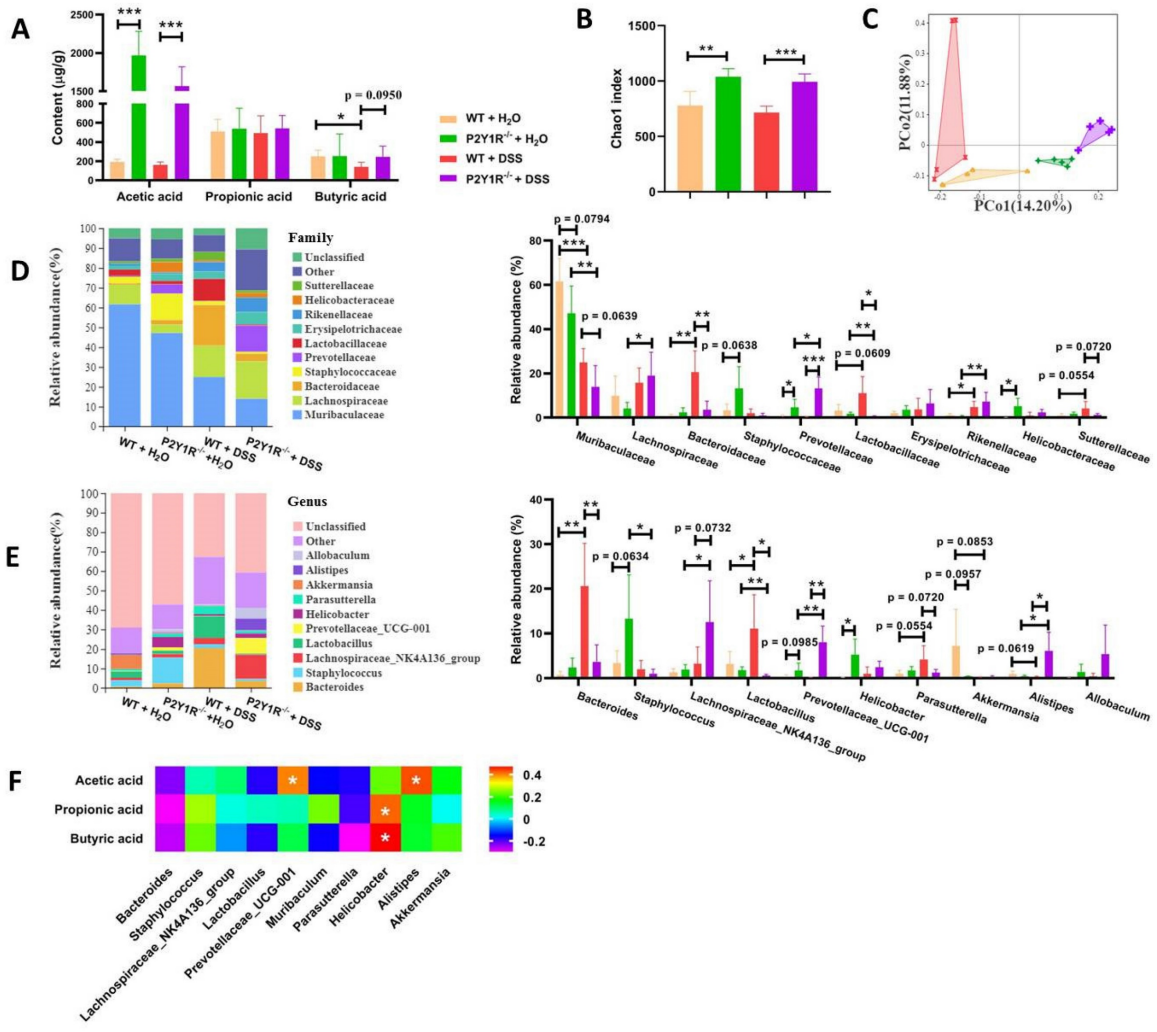
** P2Y1R inhibition alters the composition of gut microbiota and the content of fecal SCFAs**. Colitis was induced in WT or P2Y1R^-/-^ C57BL/6J mice (n = 5) for 7 days. At the end of the experiment, fresh feces were collected for SCFA content and 16S diversity analysis. (A) The contents of acetic acid, propionic acid, and butyric acid in feces. (B) The alpha diversity (Chao1 index) in four groups. (C) PCoA plots of unweighted UniFrac distances of beta diversity. (D) Relative abundance of microbiota at the family level. (E) Relative abundance of microbiota at the genus level. (F) Correlation analysis of SCFA content and microbiota at the genus level. Data represent the means ± SEM. *p < .05, **p < .01, ***p < .001.

Because gut microbiota-derived SCFAs are important guardians of intestinal epithelial integrity, we further detected changes in SCFAs in the feces of normal and colitis mice under P2Y1R inhibition. As shown in **Figure 9A**, under normal physiological conditions, P2Y1R inhibition significantly increased acetic acid production in mouse feces but had no effect on the production of propionic acid and butyric acid. During colitis, the feces of P2Y1R^-/-^ colitis mice showed higher levels of acetic and butyric acid compared with the WT colitis mice. Subsequently, the composition of the gut microbiota in the feces was analyzed using 16S diversity analysis. As shown in **Figure 9B**, the alpha diversity (Chao1 index) of P2Y1R^-/-^ mice was higher than that of the WT mice under both normal physiological and colitis conditions. The PCoA plot based on unweighted UniFrac (**Figure 9C**) showed significant separation between the four groups of microbiomes. At the family level, the fecal microbiota community was occupied by *Muribaculaceae, Lachnospiraceae, Bacteroidaceae, Staphylococcaceae,* and* Prevotellaceae*. Compared to the WT mice, P2Y1R inhibition significantly decreased the abundance of *Muribaculaceae* but increased the abundance of *Staphylococcaceae, Prevotellaceae,* and *Helicobacteraceae* under normal physiological conditions. Also, P2Y1R inhibition significantly decreased the abundance of *Muribaculaceae, Bacteroidaceae,* and* Sutterellaceae* but increased the abundance of *Prevotellaceae* under the colitis condition (**Figure 9D**). Notably, at the genus level, the fecal microbiota was occupied by the *Bacteroides, Staphylococcus,* and *Lachnospiraceae_NK4A136_group*. As shown in **Figure 9E**, P2Y1R^-/-^ mice showed higher abundances of *Staphylococcus*, *Prevotellaceae_UCG-001,* and* Helicobacter* compared with WT mice. Additionally, P2Y1R^-/-^ colitis mice showed higher abundances of *Lachnospiraceae_NK4A136_group, Prevotellaceae_UCG-001,* and* Alistipes,* and lower abundances of *Bacteroides, Lactobacillus,* and* Parasutterella* compared with WT colitis mice. A correlation analysis revealed that the concentration of fecal acetic acid was positively correlated with the abundance of *Prevotellaceae_UCG-001* and* Alistipes*, and the concentrations of fecal propionic acid and butyric acid were positively correlated with the abundance of *Helicobacter* (**Figure 9F**). Collectively, these data demonstrate that P2Y1R inhibition alters the composition of gut microbiota and the content of fecal SCFAs.

## Discussion

The intestinal mucosa is the first barrier for the host against the invasion of endotoxins, antigens, and microorganisms that potentially promote disease 29. Once this barrier is breached, invading pathogens may trigger an unfettered immune response to induce the development of IBD. Normally, IECs have the property of self-repair to maintain mucosal homeostasis, but some pathogenic factors produced in disease states may disrupt the self-repair process of IECs, leading to poor wound healing. A large number of clinical and basic studies have already confirmed that the recurrence of IBD is closely related to intestinal epithelial barrier dysfunction or insufficient mucosal wound healing 3. Therefore, it is necessary to explore the pathogenic factors and pathological mechanisms that hinder the self-repair of IECs to further understand the disease process of IBD and to develop new therapeutic strategies. In the present study, we identified that ADP released and accumulated at the site of inflammation can impede mucosal wound healing and the recovery of epithelial barrier function. We also observed that P2Y1R was highly expressed in colonic epithelial cells during colitis and found that P2Y1R inhibition could promote intestinal mucosa repair through the activation of epithelial AMPK and reconstruction of gut microbiota homeostasis.

It is well known that cells release large amounts of ATP in response to various stimuli, such as viral infection and cell damage, and the released ATP can be rapidly hydrolyzed by CD39 to ADP, maintaining the extracellular ADP concentration at a high level 30, 31. Traditionally, ATP and ADP have generally been recognized as energy currencies that play crucial roles in energy metabolism 32. In recent years, many studies have shown that ATP and ADP are potent inflammatory mediators that regulate many aspects of immune cell function, such as antigen presentation, chemotaxis, cytokine production, and lymphocyte activation, through the activation of purinergic P2 receptors 8. However, the role of ATP and ADP in regulating the mucosal barrier remains controversial. Shen et al. reported that ADP can stimulate human endothelial cell migration and promote epithelial wound healing 24. However, other studies have shown that ATP can induce the release of IL-8 in human esophageal epithelial cells 33, and ATP and ADP can also induce IL-33 and high mobility group protein 1 (HMGB1) release in primary human airway epithelial cells 34. In the present study, we identified that ADP has no significant effect on intestinal mucosal barrier function under normal physiological conditions but exhibits worsened mucosal barrier function under inflammatory conditions, which hinders intestinal epithelial wound healing and exacerbates intestinal barrier permeability by disturbing the integrity of tight junctions. In mammals, ATP can activate all P2Y receptors except P2Y6R, whereas ADP primarily activates P2Y1R, P2Y12R, and P2Y13R. Among these ADP-acting receptors, P2Y1R is highly expressed in intestinal, epithelial, and endothelial cells 8, 24. In the present study, we found that P2Y1R was significantly upregulated in the colonic epithelial cells of DSS-induced experimental colitis mice and in the colonic mucosal tissues of UC patients. Inhibition of P2Y1R with antagonist MRS2179 significantly rescued TNF-α-induced increase in the monolayer permeability and the tight junction protein claudin-2, as well as TNF-α-induced decreases in wound healing capacity and tight junction proteins claudin-1, claudin-3, ZO-1, and occludin. We also found that mouse P2Y1R deficiency in mice significantly reduced goblet cell loss and intestinal permeability; increased the levels of tight junction proteins occludin, claudin-3, and ZO-1; and decreased claudin-2 in the colon tissues of mice with colitis. These findings demonstrate that P2Y1R hinders the repair of intestinal mucosa under inflammatory conditions, such as colitis.

AMPK is an important signaling pathway involved in maintaining the health of the intestinal epithelium 25. Sun et al. reported that transfection of Caco-2 monolayers with an AMPK plasmid to enhance AMPK activity or treatment with the AMPK activator AICAR effectively reduced FITC-Dextran permeability and improved epithelial barrier function. Epithelial-specific knockout of AMPK aggravated DSS-induced colitis by damaging the integrity and ultrastructure of tight junctions 28. Olivier et al. also reported that the absence of AMPK in Caco-2 monolayers caused a delay in tight junction reorganization and repositioning during calcium switching, resulting in impaired paracellular permeability. By contrast, activation of AMPK using the pan-AMPK activator 991 accelerated reassembly and reorganization of tight junctions in Caco-2 monolayers after calcium turnover and improved paracellular permeability 35. In addition, several lines of evidence suggest that pharmacological, nutritional, and physiological activation of AMPK can strengthen epithelial apical junctions and protect the epithelial barrier from environmental stress or injury 36-39, suggesting that AMPK activation ensures better recovery of epithelial barrier function. In the present study, we found that epithelial AMPK was significantly inactivated in an inflammatory environment, and ADP release at the site of inflammation enhances epithelial AMPK inactivation induced by inflammatory factors such as TNF-α. Notably, inhibition of P2Y1R significantly reversed this inactivation, and inhibition of AMPK signaling with siRNA significantly attenuated the beneficial effects of P2Y1R inhibition on wound healing and restoration of epithelial barrier function. Similarly, the same observation has been well validated *in vivo*. Collectively, these findings demonstrate that P2Y1R regulates intestinal mucosal repair in an AMPK-dependent manner.

It is well known that bacteria-epithelial cell interactions play an essential role in maintaining the health of the intestinal epithelium 40. In the gastrointestinal tract, IECs maintain homeostasis between the microbiome and the host by constructing a mucus barrier and initiating humoral immune responses 40. At the same time, metabolites produced by microorganisms, such as SCFAs, regulate lumen pH and mucus production, modulate intracellular signals of epithelial cells, and provide fuel for epithelial cells that are critical for intestinal integrity 41. For example, microbial-derived acetate can induce chromatin remodeling in IECs, coregulating host metabolism and intestinal innate immunity through the conserved tip60 steroid hormone axis 42. Microbial-derived butyric acid can also enhance MUC2 expression, activate the MUC2 promoter, and enhance histone acetylation in cell cultures by inhibiting HDAC 43. Once the microbial environment changes, its metabolites also change accordingly, disrupting the bacteria-epithelial cell interaction, thereby damaging the integrity of the intestinal epithelium and leading to short- and long-term pathological changes in the host 40. In the present study, we found that P2Y1R inhibition significantly increased the abundance of *Prevotellaceae_UCG-001*, *Parasutterella,* and *Helicobacter*, while decreasing the abundance of *Alistipes* under normal physiological conditions. In addition, under colitis conditions, P2Y1R inhibition also increased the abundance of *Lachnospiraceae_NK4A136_group*, *Prevotellaceae_UCG-001,* and *Alistipes*, while decreasing the abundance of *Bacteroides*, *Lactobacillus,* and *Parasutterella*. In addition, P2Y1R inhibition also increased fecal acetic acid production under normal physiological conditions, and fecal acetic and butyric acid productions under colitis conditions. These data demonstrated that P2Y1R inhibition had significant effects on the composition of gut microbiota and SCFA production. Previous studies reported that *Prevotellaceae_UCG-001* 44, 45 and *Alistipes* 46, 47 were SCFA-producing bacteria. In the present study, our data showed that fecal acetic acid production was positively correlated with the abundance of *Prevotellaceae_UCG-001* and *Alistipes*, and fecal propionic acid and butyric acid were positively correlated with the abundance of *Helicobacter*. To date, no studies have confirmed that bacteria from *Helicobacter* are SCFA-producing bacteria, but it has been reported that *Helicobacter hepatis* from *Helicobacter* can induce host cells to secrete potent IL-10 to counteract the pathogen-associated molecular pattern-mediated proinflammatory effects of bacteria on the immune system 48, suggesting that there may be a potential link. Notably, in both normal physiological and colitis conditions, P2Y1R inhibition can increase the relative abundance of *Prevotellaceae_UCG-001* and its metabolite acetic acid. Recently, Deleu et al. reported that acetate can protect the intestinal barrier and exert anti-inflammatory effects in organoid-derived monolayer epithelial cultures in patients with UC 49. Taken together, these results suggest that P2Y1R inhibition also mediates intestinal mucosal repair by regulating SCFA-producing bacteria, particularly *Prevotellaceae_UCG-001*.

Although this study revealed some important findings, there are still some limitations. This study focused on the role of epithelial P2Y1R in intestinal mucosa repair. Although existing data indicate that epithelial P2Y1R impedes intestinal mucosa repair under inflammatory conditions, additional experiments in epithelial-specific P2Y1R knockout mice are needed to confirm these results. In addition, in exploring the mechanism of P2Y1R inhibition promoting intestinal mucosal repair, it was identified that P2Y1R inhibition might mediate intestinal mucosal repair by regulating SCFA-producing bacteria, particularly *Prevotellaceae_UCG-001*. The role of *Prevotellaceae_UCG-001* in promoting intestinal mucosal repair through acetic acid, its metabolite, remains to be further verified.

In summary, the present study demonstrated that P2Y1R was highly expressed in colonic epithelial cells during colitis, and ADP, an endogenous ligand released at the site of inflammation, could activate P2Y1R and hinder intestinal mucosa repair, while inhibition of P2Y1R could promote intestinal mucosal repair by activating epithelial AMPK and reestablishing intestinal flora homeostasis. This study highlights the functional role of P2Y1R in regulating intestinal barrier homeostasis. Combined with the role of P2Y1R in immune inflammation, visceral sensitivity, and intestinal motility, P2Y1R is a potential target for the treatment of IBD, and P2Y1R antagonists may serve as effective drug candidates for IBD therapy.

## Figures and Tables

**Table 1:** Corresponding clinical data of selected healthy subjects and UC patients

Characteristics	Normal control (n = 20)	Inactive UC (n= 7)	Active UC (n = 34)
**Gender**			
Male	12	3	20
Female	8	4	14
**Age (years ± SD)**	42.35±11.17	40.86±5.33	41.2±13.58
**Extent of disease**			
Extensive colitis		0	9
Sided colitis		4	13
Proctitis		3	12
**Treatment**			
Amino salicylates		6	27
Corticosteroids		0	1
None		1	6
**Endoscopy**			
Grade 0-1		7	9
Grade 2-3		0	25

**Table 2:** Real-Time PCR Primer Sequences

Gene Name	Primer Sequences
Mouse GAPDH	Forward 5′-GGTTGTCTCCTGCGACTTCA-3′
	Reverse 5′-TGGTCCAGGGTTTCTTACTCC-3′
Mouse Occludin	Forward 5′-TTGGCTACGGAGGTGGCTATGG-3′
	Reverse 5′-TTACTAAGGAAGCGATGAAGCAGAAGG-3′
Mouse Claudin-3	Forward 5′-ACCGTACCGTCACCACTACCAG-3′
	Reverse 5′-AGATGCCAGCCTGTCTGTCCTC-3′
Mouse ZO-2	Forward 5′-CATGTCTCTAACGGATGCTCGGAAG-3′
	Reverse 5′-GTTTAGGGCTGGGATGTTGATGAGG-3′
Mouse Claudin-2	Forward 5′-CACCGTGTTCTGCCAGGATTCTC-3′
	Reverse 5′-GATAAAGCCCAGGATGCCACCAAG-3′
Human GAPDH	Forward 5′-CAGGAGGCATTGCTGATGAT-3′
	Reverse 5′- GAAGGCTGGGGCTCATTT-3′
Human Claudin-1	Forward 5′-AGGTGCTGCTGAGGATAGACTGAC-3′
	Reverse 5′-TGGCGAGCATCCTTGGCAATTC-3′
Human ZO-1	Forward 5′-GCATGATGATCGTCTGTCCTACCTG-3′
	Reverse 5′-CCGCCTTCTGTGTCTGTGTCTTC-3′
Human Claudin-3	Forward 5′-TCATCGGCAGCAACATCATCACG-3′
	Reverse 5′-CAGCAGCGAGTCGTACACCTTG-3′
Human Claudin-2	Forward 5′-AGGTGCTGCTGAGGATAGACTGAC-3′
	Reverse 5′-TGGCGAGCATCCTTGGCAATTC -3′
